# Strategies for Accurate Cell Type Identification in CODEX Multiplexed Imaging Data

**DOI:** 10.3389/fimmu.2021.727626

**Published:** 2021-08-13

**Authors:** John W. Hickey, Yuqi Tan, Garry P. Nolan, Yury Goltsev

**Affiliations:** ^1^Department of Microbiology and Immunology, Stanford University School of Medicine, Stanford, CA, United States; ^2^Department of Pathology, Stanford University School of Medicine, Stanford, CA, United States

**Keywords:** Multiplexed tissue imaging, CODEX, single-cell analysis, normalization, unsupervised clustering, spatial analysis, cell-type identification, colon

## Abstract

Multiplexed imaging is a recently developed and powerful single-cell biology research tool. However, it presents new sources of technical noise that are distinct from other types of single-cell data, necessitating new practices for single-cell multiplexed imaging processing and analysis, particularly regarding cell-type identification. Here we created single-cell multiplexed imaging datasets by performing CODEX on four sections of the human colon (ascending, transverse, descending, and sigmoid) using a panel of 47 oligonucleotide-barcoded antibodies. After cell segmentation, we implemented five different normalization techniques crossed with four unsupervised clustering algorithms, resulting in 20 unique cell-type annotations for the same dataset. We generated two standard annotations: hand-gated cell types and cell types produced by over-clustering with spatial verification. We then compared these annotations at four levels of cell-type granularity. First, increasing cell-type granularity led to decreased labeling accuracy; therefore, subtle phenotype annotations should be avoided at the clustering step. Second, accuracy in cell-type identification varied more with normalization choice than with clustering algorithm. Third, unsupervised clustering better accounted for segmentation noise during cell-type annotation than hand-gating. Fourth, Z-score normalization was generally effective in mitigating the effects of noise from single-cell multiplexed imaging. Variation in cell-type identification will lead to significant differential spatial results such as cellular neighborhood analysis; consequently, we also make recommendations for accurately assigning cell-type labels to CODEX multiplexed imaging.

## Introduction

Multiplexed imaging techniques allow imaging up to 60 markers in a tissue simultaneously, which increases the number of identifiable cell types *in situ* (1–3). This enables a level of spatial analysis of cells that not possible using other immunophenotyping approaches (4, 5). Spatial and structural relationships are now at the forefront of biological, consortia-led, and clinical studies using these technologies (6–10). However, these multiplexed imaging technologies have unique sources of noise: imperfect cell segmentation, image processing artifacts, and tissue processing artifacts like autofluorescence (2, 11–14).

Although not problematic for qualitative analysis, these sources of noise can interfere with quantitative single-cell analysis—particularly cell-type identification. Incorrect cell-type identification will lead to false interpretations of spatial features and study conclusions. Most studies using multiplexed imaging technologies have employed previously established pipelines created for non-imaging-based, single-cell-type identification, such as hand-gating flow plots or unsupervised clustering, and have used various methods of raw data processing and normalization (10, 15–20).

Here we describe a study benchmarking the effects of normalization techniques and unsupervised clustering algorithms on multiplexed imaging data. In this study, we evaluated the performance of five major normalization techniques and four unsupervised clustering algorithms on mitigating the effects of noise in cell-type identification in a dataset generated by the co-detection by indexing (CODEX) multiplexed imaging technology.

## Materials And Methods

### CODEX Imaging

CODEX multiplexed imaging was done using a CODEX staining and imaging protocol previously described in detail (16, 19). Settings used for the microscope are listed in **Supplemental Table 1**. The 47 antibodies were custom conjugated to oligonucleotides following the published protocol. Antibody information is summarized in **Supplemental Table 1**. Raw imaging data were then processed using the CODEX Uploader for image stitching, drift compensation, deconvolution, and cycle concatenation. Processed data were segmented using the CODEX Segmenter, a watershed-based single-cell segmentation algorithm. Both the CODEX Uploader and Segmenter are software can be downloaded from our GitHub site (https://github.com/nolanlab/CODEX).

### Normalization Techniques

We compared single-cell quantified data without processing to that processed using four different normalization techniques:

#### Z Normalization

Each marker intensity was Z normalized separately for all cells within the dataset. This normalized the range of each marker as fluorescent intensities of each marker can depend on antibody staining strength and exposure times.

#### Log (Double Z) Normalization

The first Z normalization was performed on each marker intensity, and then another Z normalization was applied to each cell. These values were then transformed into probabilities. Finally, a negative log transformation was applied to the complement of the probabilities. Because the first Z normalization equalizes signal intensities, marker Z scores can be compared. Furthermore, as each cell should only be positive for between one and five markers of the 47 recognized by antibodies in the staining panel, applying the second Z normalization identifies positive markers with high probability. Using a negative log transformation of the complement of the probability is necessary to amplify values of high probabilities for input into clustering algorithms.

#### Min_Max Normalization

First the 1^st^ and 99^th^ percentiles were found to cap minimum and maximum values, respectively, for each fluorescent channel and then each value in the channel was normalized by taking the difference between minimum over the range of values. Reducing to the 99^th^ percentile aids removes artificially high background fluorescent intensities often seen in imaging datasets.

#### Arcsinh Normalization

An arcsinh transformation was performed on marker intensities, and the resulting values were scaled with a cofactor of 150. This type of normalization is appropriate when dataset contain low or negative values resulting from background subtraction.

### Unsupervised Clustering Techniques

Hand gating was carried out using a hierarchical strategy to label each cell as shown in **Supplemental Figure 1** using CellEngine (https://cellengine.com/). X-shift with angular distance, X-shift with Euclidian distance, and *k*-means clustering were performed using the VorteX software available from our GitHub site (https://github.com/nolanlab/vortex). Default settings were used with *k* values obtained from the elbow-point inflection from each clustering technique. Leiden-based clustering was performed using the scanpy Python package with default parameters.

### F-Score and Neighborhood Analysis

F-score analysis was performed as described in **Figure 3A** using the indicated reference dataset for each comparison. Neighborhood analysis was performed using the same Python scripts described previously (10). Neighborhoods were named for cell types enriched within the neighborhood as compared to the tissue as a whole (**Supplemental Figure 12**).

## Results

### CODEX Multiplexed Imaging of the Human Colon

**Figure 1:**
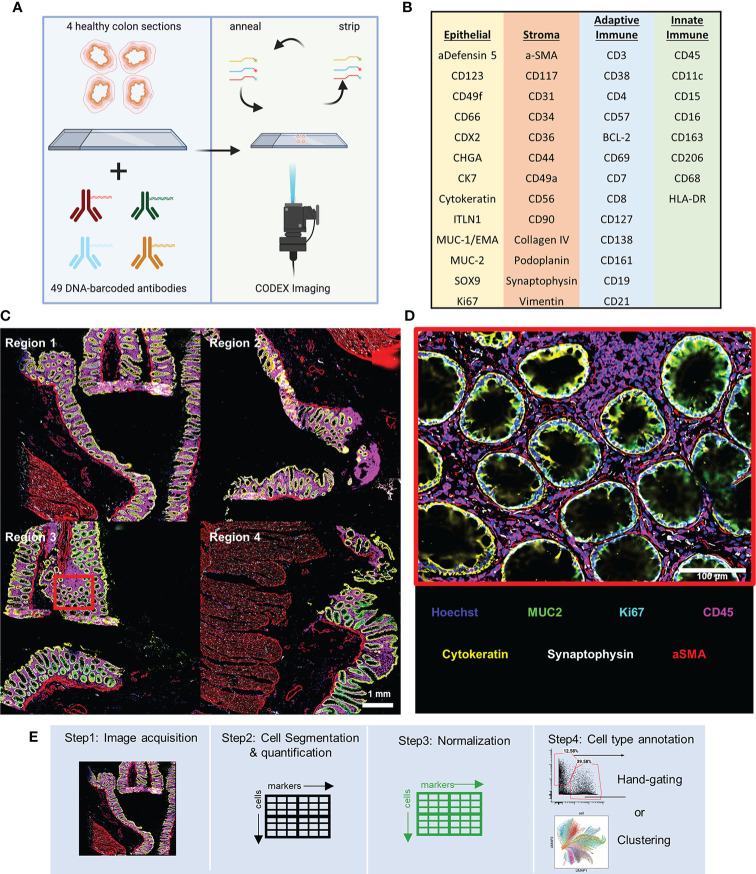
CODEX multiplexed imaging of the human colon. **(A)** Schematic of the CODEX protocol for imaging of sections of ascending, transverse, descending, and sigmoid colon. **(B)** The 47 CODEX oligonucleotide-barcoded antibodies used discriminate several major epithelial subtypes of the colon, stromal cell types, and both adaptive and innate immune cell types. **(C)** Image with six representative markers highlighted with data for CD45 (magenta), MUC2 (green), Ki67 (cyan), Synaptophysin (gray), Cytokeratin (yellow), and aSMA (red) (scale bar = 1 mm). **(D)** Magnified view of the region indicated with the cyan box in panel **(C)** (scale bar = 100 μm). **(E)** The workflow for single-cell multiplexed imaging preprocessing used in this study.

We conducted our analysis on data we collected as a part of the Human BioMolecular Atlas Program (HuBMAP) consortia effort that focuses on systematic mapping healthy tissue structure across human organ systems and making the data publicly available (6). For this analysis we used imaging data collected from four tissue blocks from the same human donor from the transverse, ascending, descending, and sigmoid colon (regions 1-4, respectively) made into a single array. We used a 47 oligonucleotide-barcoded antibody panel to image with the CODEX technology (16, 19), which involves cyclic stripping, annealing, and imaging of fluorescently labeled oligonucleotides complementary to the oligonucleotides that barcode the antibodies used in staining (**Figure 1A**).

The antibody panel includes targets for discriminating several major epithelial subtypes of the colon, stromal cell types, and both adaptive and innate immune cell types (**Figure 1B**). Using only six markers we can observe major cell subsets of the colon: immune (magenta), goblet (green), proliferating (cyan), nerve (gray), general epithelial (yellow), and smooth muscle (red) cells (**Figures 1C, D** **)**.

CODEX imaging of the colon tissue resulted in a single-cell dataset composed of ~130,000 cells with fluorescence values quantified from each marker by standard processing of CODEX imaging data: tile stitching, drift compensation, cycle concatenation, background subtraction, deconvolution, determination of best focal plane, and segmentation of single cells ready for cell type annotations (**Figure 1E**).

### Methods for Normalization and Unsupervised Clustering Techniques Used for Cell Type Identification

**Figure 2:**
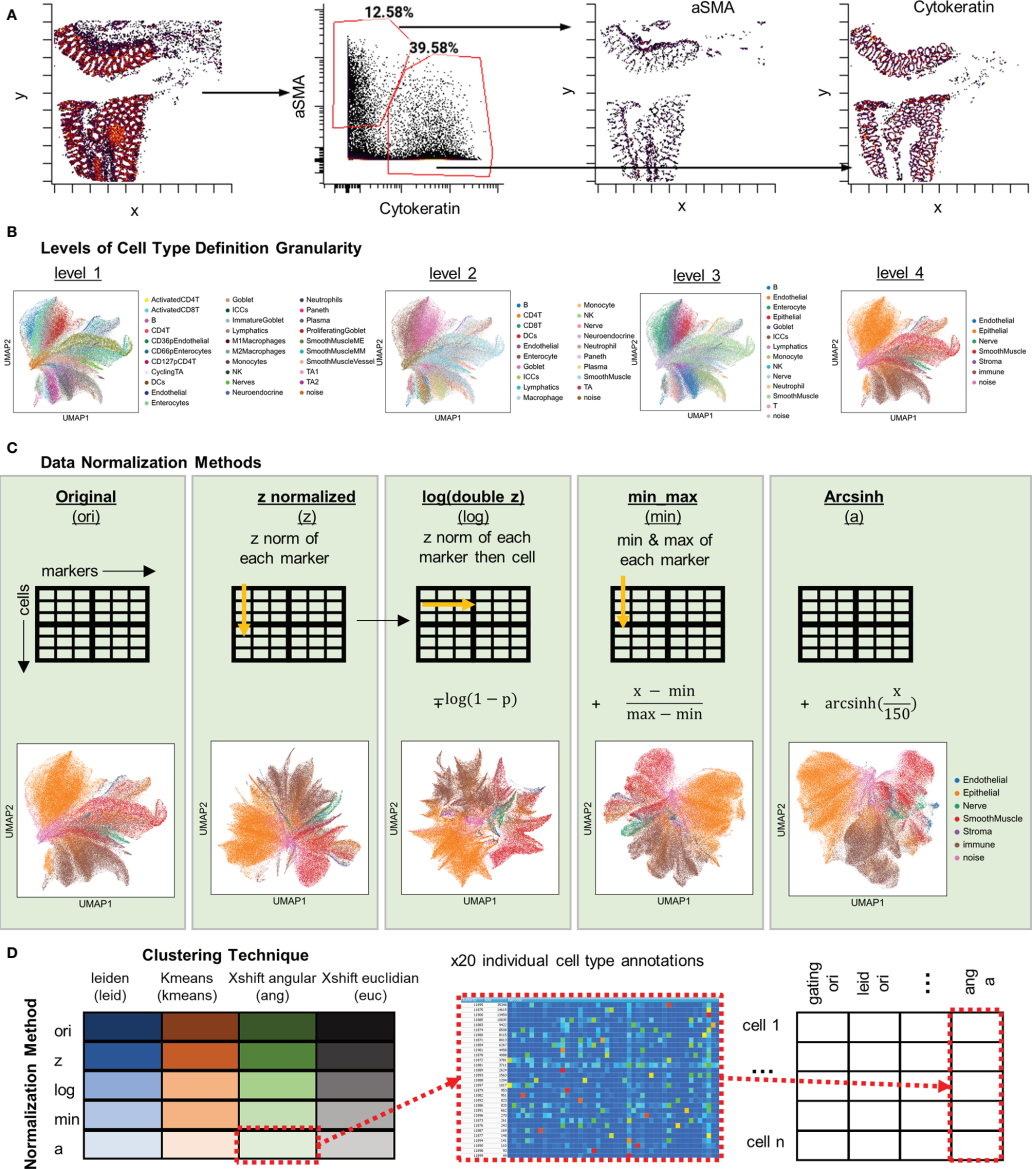
Strategies used for cell type annotation *via* five methods of data normalization and four clustering algorithms. **(A)** Far left: Spatial plot of x, y positions cells gated based on quantified fluorescent signal for aSMA and Cytokeratin. Plots to the right: Single-positive gating shows location of identified populations with the same x, y positions. **(B)** Cell-type comparisons at four levels of granularity with the level 1 having the highest degree of granularity of 35 cell types and level 4 having the lowest degree of granularity of 7 types. **(C)** Schematic of data treatment and representative UMAP plots for, from left to right, original CODEX data, Z normalized data, log(double Z) transformed data, min-max normalized data, and arcsinh normalized data. **(D)** Data, normalized or not, was clustered using the Leiden algorithm, *k-*means, X-shift with Euclidian distance, or X-shift with angular distance. All cell type annotations were merged for each cell and combined into one dataset for comparison of annotations.

We first used the hand-gating approach to hierarchically gate out 35 distinct cell types in the dataset (**Figures 2A, B** and **Supplemental Figure 1**). Hand gating is often used for cell type identification in immunophenotyping techniques like flow or mass cytometry and is an often used gold-standard for comparison of cell-type annotations (21). In addition to gating out marker values, with CODEX data we can also visualize or gate on the spatial location of cells based on markers (**Figure 2A**).

Since the granularity of cell-type definition is dependent on the data analyzer, we also explored the confidence of cell-type discrimination with multiplexed imaging data. To do this, we defined four levels of cell-type granularity: level 1, 35 cells types (including a “noise” cell type); level 2, 20 cell types; level 3, 14 cell types; and level 4, 7 cell types (**Figure 2B**). Discrimination at the highest level of granularity should provide the most detailed understanding of tissue biology, but increasing granularity comes at a cost of confidence in accuracy.

To compare the influence of normalization techniques, the quantified fluorescent data was subjected to one of four normalization techniques: Z, log(double Z), min-max, or arcsinh, or left in its original raw format (**Figure 2C**). The transformed data were then used as input to four different unsupervised clustering algorithms: Leiden (graph-based), X-shift (density-based) with either Euclidian or angular distances, and *k*-means (**Figure 2D**). This produced 20 separate clusterings, with each cell annotated based on cluster membership, that could be directly compared to each other and to the hand-gated standard.

### Comparison of Normalization and Unsupervised Clustering Techniques to Hand-Gated Cell Type Annotations

**Figure 3:**
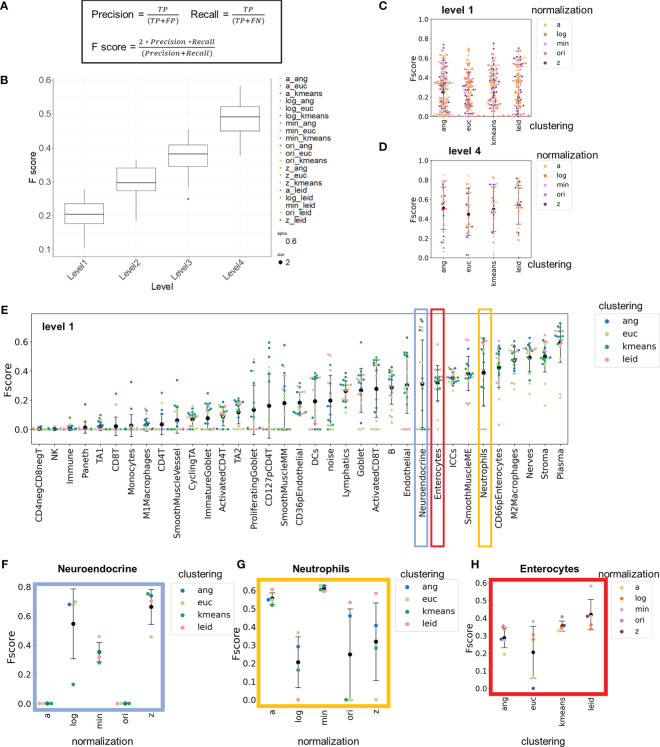
F-score comparisons between clustering and normalization technique to hand-gated standard demonstrate high inter-cellular and high intra-cellular variation. **(A)** Method for calculating F-scores with cell-type assignments. **(B)** Average F1 score for each normalization. **(C)** Clustering combinations stratified by level of granularity. **(D)** Level 1 and level 4 F-scores averaged across cell types for each combination of normalization technique and clustering algorithm. **(E–H)** Level 1 F-scores for **(E)** each cell type and, in expanded views, **(F)** neuroendocrine cells, **(G)** neutrophils, and **(H)** enterocytes pulled out for comparisons of clustering or normalization technique (black data point is mean and error bars indicate standard deviation).

All percentages of cell types were fairly similar across with values within 10% for all cell type annotations (**Supplemental Figure 2**) and numbers of unique cell types identified were similar across the clusterings (**Supplemental Figure 3**). To compare annotations more statistically between the unsupervised and hand-gated populations, the F-score was calculated for all clustering algorithms and normalization combinations at each cell type. The F-score is a commonly used metric to refer to the concordance of a prediction and a gold standard and is defined as the harmonic mean of the precision and recall (**Figure 3A**, **Supplemental Figure 4**). This metric is widely used because it considers false positives and false negatives. The F-score ranges from 0 to 1 where 0 is no concordance and 1 is perfect accuracy between the gold standard and the predication.

F-scores summarized over all cell types revealed that the highest level of granularity in cell-type identification had a low-level agreement with the gold-standard hand gating (0.1-0.3, **Figures 3B, C** **)** with high variation. The F-score average increased (to 0.4-0.6) at higher levels of granularity (**Figures 3B, D** and **Supplemental Figure 5**). As expected, given the variation in F-scores, grouping of the data on a per cell type basis revealed stark inter-cell and intra-cell variation of F-scores (**Figure 3E**).

Understanding the inter-cell variation of F-scores can help us gauge the appropriate granularity for cell-type assignment. Certain cell types (e.g., interstitial cells of Cajal, plasma, stromal cells) were consistently categorized accurately (average F-score of 0.6), whereas other cell types (e.g., CD4^-^ CD8^-^ T cells, CD4^+^ T cells, CD127^+^ pCD4^+^ T cells) were not (average F-score of 0.05), regardless of normalization technique or clustering algorithm used.

A major reason certain cell types have consistently low F-scores is that these types of cells were not identified by many of the approaches (**Supplemental Figure 6**). Another reason is that the granularity of cell type definition at level 1 includes the use of phenotypic markers to split cell types. For example, the identification of CD4^+^ T cells at level 1 is poor because CD4^+^ T cell subpopulations were based on CD127 and CD69 expression. Although these distinct cell types were evident in many clustering datasets based on average expression profiles, the intensity profiles of single-cell data are a continuum rather than binary, characteristic of many phenotypic markers like CD69 and CD127 (**Supplemental Figure 7A**). This makes it harder to consistently resolve cell types based on phenotypic markers. The next level of granularity, level 2, eliminates these phenotypic separations, and CD4^+^ T cells were one of the more consistently recognized cell types (F-score increased from 0.05 with level 1 granularity to 0.5 at level 2) (**Supplemental Figure 7B**). Consequently, phenotypic definitions of cell types should be avoided in cell-type annotations.

We verified these general trends using another quantitative metric, Cohen’s kappa, which is a measure of chanced corrected accuracy. Cohen’s kappa ranges from 0 to 1 with higher scores indicative of better agreement between two labels. Cohen’s kappa was also inversely correlated with the granularity of the cell type (**Supplemental Figure 8A**). This metric demonstrated that X-shift clustering with Euclidian distance clustering performed poorly regardless of normalization technique (**Supplemental Figures 8B, C**). The F-scores and the Cohen’s kappa scores were correlated indicating agreement within the two metrics (**Supplemental Figure 8D**).

Understanding intra-cell variation of F-scores informs how different combinations of clustering algorithms and normalization techniques influence cell-type labeling accuracy for CODEX data. For some cell types (e.g., neuroendocrine cells and neutrophils) there was high intra-cell variation of F-scores (~0.7 range), whereas for other cell types (e.g., enterocytes) there was considerably less intra-cell variation of F-scores (~0.15 range). We highlight three examples with both high and low intra-cell variation are instructive of the differences in normalization and clustering techniques.

Neuroendocrine cells are a rare cell type (~0.3% of all cells) and are uniquely positive for chromogranin A (CHGA). CHGA had a low signal intensity in the fluorescence but also had a low background signal (**Supplemental Figure 9A**). F-scores were largely dependent on normalization technique and not on the unsupervised clustering algorithm (**Figure 3F** and **Supplemental Figure 9B**). When Z normalization was applied, these cells were consistently identified (F-score of 0.65); in contrast, without normalization, these cells were not identified (F-score of 0).

Neutrophils are also rare (~0.3% of all cells) and are identified due to expression of CD45, CD15, and CD16, which are markers also shared by other immune and epithelial cells. CD15 and CD16 had higher background signals than other markers (**Supplemental Figure 9C**), and the F-scores were dependent on the normalization technique (**Figure 3G** and **Supplemental Figure 9D**). The min-max and arcsinh normalizations had high consistency (<0.1 F-score range) regardless of the clustering algorithm, whereas F-scores determined with other normalization techniques varied widely and depended on the downstream clustering algorithms (~0.5 F-score range).

Enterocytes are a common cell type (~12% of all cells) identified based on cytokeratin staining and lack of specialized epithelial markers (e.g., MUC2). The intra-cell F-score variation was low for these cells. Differences between normalization and clustering technique were less pronounced than for neuroendocrine cells and neutrophils, although for enterocytes agreement depended more on the clustering technique than for rarer cells types. For enterocytes, Leiden and *k*-means clustering had more consistent F-scores than did X-shift-based methods (**Figure 3H** and **Supplemental Figure 9E**).

It is important to select the normalization and clustering technique that maximizes the performance on the cell types (e.g., neuroendocrine cells and neutrophils) that have high intra-cell variation of F-scores. Our analysis indicates that a normalization method such as Z normalization reduces clustering artifacts associated with noise associated with low signal or high background.

### Hand-Gating Annotations Are Limited by Segmentation Noise

**Figure 4:**
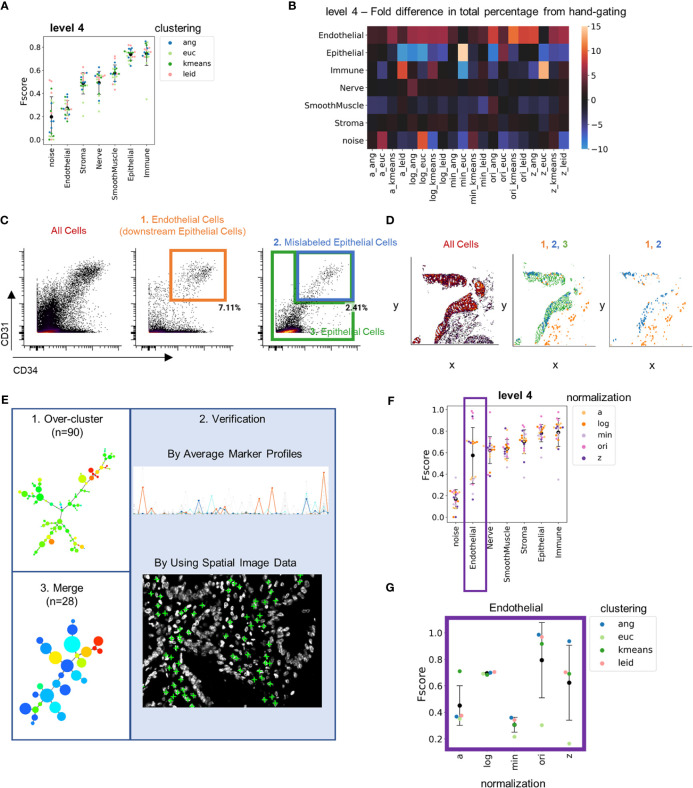
Hand-gating is confounded by cell segmentation noise. **(A)** Level 4 F-score averaged across cell types for each combination of normalization technique and clustering algorithm as compared to hand-gating standard. **(B)** Heatmap of fold differences between cell-type percentages for level 4 for each combination of normalization technique and clustering algorithm as compared to hand gating annotations. **(C)** CD34 versus CD31 fluorescent intensity (endothelial markers) for, from left to right, all cells, actual endothelial cells that are downstream of the Cytokeratin gate (orange box), and both mislabeled epithelial cells (blue) and all other epithelial cells (green). **(D)** Plots of, from left to right, all gated cells, three populations indicated by orange, green, and blue boxes in panel **(C)**, and just endothelial and mislabeled epithelial cells. **(E)** Overview of technique to generate an over-clustered standard dataset: 1) 90 clusters were generated by X-shift angular distance clustering with the original cell data, 2) clusters were annotated with cell type by evaluation of marker average profiles and location of cells withing tissue, and 3) common cell types were merged to a final standard dataset of 28 clusters. **(F)** Level 4 F-scores averaged across cell types for each combination of normalization technique and clustering algorithm as compared to over-clustered standard. **(G)** Level 4 F-scores for the endothelial cell population for combinations of normalization and clustering techniques compared to the over-clustered standard (black data point is mean and error bars indicate standard deviation).

F-score averages increased at lower levels of granularity as expected, although, surprisingly, the endothelial cell population had a consistent low average score (**Figure 4A**). We investigated this by computing the fold change of cell-type percentage from each clustering result compared to the hand-gated standard (**Figure 4B** and **Supplemental Figure 10A**). Endothelial cells were identified more frequently (5- to 10-fold higher total numbers) by clustering annotation compared to hand gating. In contrast, there were fewer epithelial cells (5- to 10-fold fewer total numbers) identified by clustering than by hand-gating assignment. This suggests that the hand-gated standard may have incorrectly misclassified endothelial cells as epithelial cells. In the hand-gating process, endothelial cells were identified as cells that were negative for cytokeratin and positive for CD34 and CD31 (**Figure 4C**). Only 2% of cells that expressed cytokeratin were also positive for CD34 and CD31 (**Figure 4C** and **Supplemental Figure 10B**).

Imperfect cell segmentation often happens in regions where cells are in close proximity and are not separated. This contributes to the noise in multiplexed imaging data. To understand if this might be the reason for mislabeling of endothelial cells, we looked at the spatial locations of endothelial cells, the mislabeled epithelial cells, and the epithelial cells that were defined by hand gating. The mislabeled endothelial cells were located closely adjacent to the epithelium (**Figure 4D**). This indicates that cells were misassigned due to segmentation noise in locations where the cytokeratin stain bleeds into endothelial cell populations. Because hand gating only can segregate cell types hierarchically using two markers at a time, this strategy cannot deal with segmentation noise well.

Since hand-gating cell type identification does not handle cell segmentation noise well, we required a new gold standard. To create this standard, the original data was over-clustered into 90 clusters using X-shift clustering with angular distance (**Figure 4E**). We used X-shift clustering with angular distance as this approach was accurate across levels of granularity (**Supplemental Figure 8C**). Over-clustering the data enhanced separation of cell types often confounded by noise into distinct clusters and overlaying these clusters on imaging data enabled expert users to determine accurate cell-type annotations based on staining and morphology. Clusters were also classified and merged using average cluster profiles, resulting in identification of 28 unique cell types.

Comparing the clustering outputs to this new gold-standard annotation substantially increased the average F-score of endothelial cells at level 4 granularity from 0.2 to 0.6 (**Figure 4F**). However, there was still high variation between clustering outputs for endothelial cells. Isolating the endothelial cells at level 1 revealed that the accuracy of cell-type annotation was more dependent on normalization technique than clustering technique. Z normalization and log(double Z) normalizations provided more consistent performance than did min-max or arcsinh normalizations (**Figure 4G** and **Supplemental Figure 11**). This result further emphasizes the importance of CODEX data normalization prior to clustering.

### Cross Comparison of Normalization and Unsupervised Clustering Techniques

**Figure 5:**
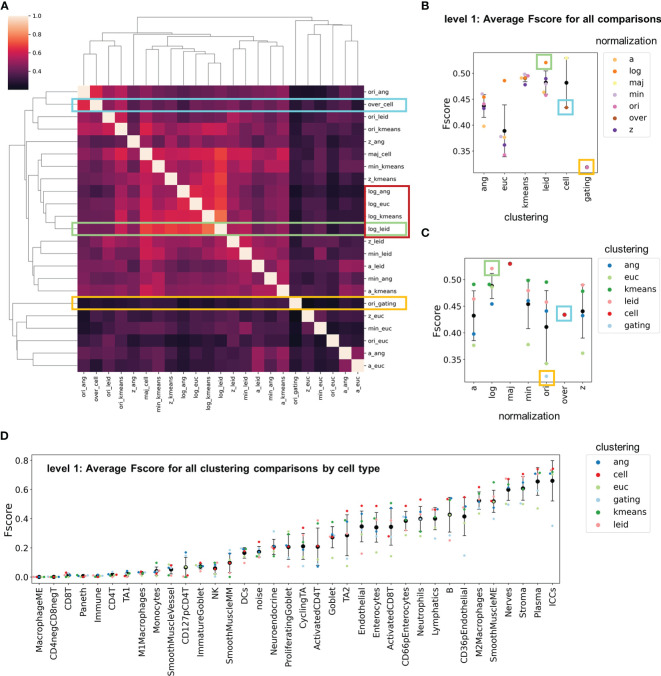
F-score comparisons between all clustering and normalization techniques as standards. **(A)** Clustered heatmap with F-scores averaged across cell types. The yellow rectangle indicates the comparisons made in **Figure 3**, the cyan rectangle indicates the comparisons made in **Figure 4**, and the green rectangle indicates the comparisons made when log double Z normalization combined with Leiden clustering was used as the gold standard. The red rectangle indicates exemplary similarities between normalization techniques. **(B, C)** Level 1 F-scores averaged over cell types for **(B)** clustering and **(C)** normalizations for all comparisons shown in panel **(A, D)** Level 1 F-scores averaged over cell types for all normalization and clustering combinations (black data point is mean and error bars indicate standard deviation).

In general, the over-clustering annotations demonstrated a bias towards agreement with X-shift angular clustering and original untransformed data as expected since these were the conditions used to generate the annotations (**Supplemental Figure 12**). To understand the extent of inter-agreement between choices of clustering algorithm and normalization technique, we used each individual clustering result as the gold standard for comparison. We first averaged the F-scores for each clustering output and cell type. Similar to previous observations, the normalization method dominated similarity between combinations of clustering and normalization algorithms (**Figure 5A**).

By further averaging all F-scores across these comparisons we identified normalization techniques and clustering algorithms that resulted in the greatest variation in cell-type assignment (**Figures 5B, C** **)**. Hand-gated assignments had the lowest overall average, which confirms that this method is not ideal for CODEX multiplexed imaging assignment. In clustering algorithm comparisons, X-shift with Euclidian distance had the highest variance and lowest F-score average (**Figure 5B**). Furthermore, *k*-means and Leiden clustering algorithms had the highest consistency and least variation (**Figure 5A**).

We also averaged F-scores to compare all combinations for each cell type (**Figure 5D**). The results are consistent with those of individual comparisons (compare to **Figure 3E**). Certain cell types are consistently recognized accurately (e.g., interstitial cells of Cajal, plasma cells) and others are not assigned accurately (e.g., CD4^+^ T cells, CD127^+^ pCD4^+^ T cells). This strengthens the argument that phenotypic cell-type calling should be limited in initial clustering due to the low confidence of these cell-type definitions (**Supplemental Figure 13**).

### Neighborhood Analysis Reveals Sources of Noise in Cell-Type Calling

**Figure 6:**
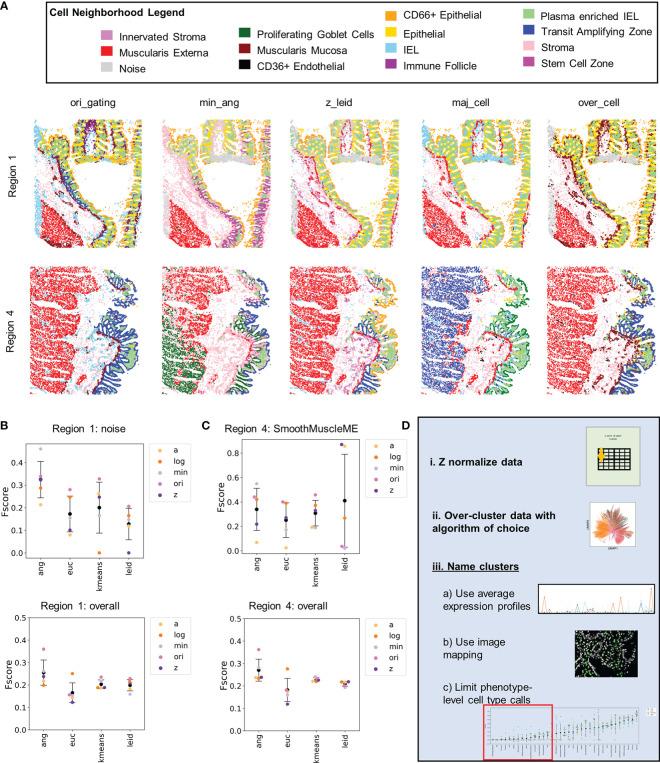
Cellular neighborhood analysis reveals additional noisy cell populations that can be managed by data normalization. **(A)** Cellular neighborhoods shown for Region 1 and 4 for five of the 23 cell-type annotations. **(B)** Level 1 F-score averages for Region 1 for the noise cell type and averaged across all cell types. **(C)** Level 1 F-score averages for Region 1 for the smooth muscle muscularis externa cell type and averaged across all cell types (black data point is mean and error bars indicate standard deviation). **(D)** Recommendations for normalization and cell type annotations of segmented single-cell CODEX data.

CODEX multiplexed data can be used to spatially map cell types and to characterize cell neighborhoods (10). We identified cell neighborhoods for five exemplary annotations at the highest level of cell-type granularity (**Supplemental Figure 14**). Visual inspection revealed that some neighborhoods (e.g., plasma-enriched interstitial epithelial lymphocytes) are present in all regions of the colon, whereas there was considerable variation in other neighborhoods (e.g., transit amplifying zone) (**Figure 6A** and **Supplemental Figure 15**). Particularly noticeable are the differences between muscularis externa and noise (enriched for noise cells) neighborhoods. Both neighborhoods primarily depend on one cell type: noise and smooth muscle muscularis externa, respectively.

Imaging noise was particularly located within the tissue sample from the transverse colon (region 1) as there were areas where the tissue folded during the cutting process, and some edge effects that were noticeable in the folded area (**Supplemental Figure 16A**). Focusing on this region, the F-scores for noise depended more on clustering algorithm than normalization; use of X-shift angular distance clustering resulted in the highest average F-score (**Figure 6B** and **Supplemental Figure 16B**). This result closely mirrors the trend of the ability of clustering algorithm to identify higher numbers of unique cell types (**Supplemental Figure 3**). This suggests the need for overclustering of CODEX multiplexed data to segregate out noise from the cell-type clusters.

Hand-gating did not accurately pick up the noise in the folded region and labeled many cells in this area as immune cell types. This is another limitation of the hand-gating approach. Even though no actual noise cells were identified by the Z normalization and Leiden combination, this combination did identify the noise neighborhood. This demonstrates the ability of this combination of normalization and clustering to recognize noise at later stages of the data analysis pipeline.

Smooth muscle muscularis externa had significant background signal from the MUC2 channel in tissue from the sigmoid colon (region 4). Smooth muscle cells were often incorrectly assigned as cells of goblet or epithelium origin as demonstrated by assignment to the muscularis externa neighborhood (**Supplemental Figure 16C**). Only Leiden clustering with either Z or arcsinh normalizations were able to accurately eliminate noise and accurately assign the majority of the smooth muscle cells (**Figure 6C** and **Supplemental Figure 16D**). This demonstrates that cell-type assignment without image verification can lead to faulty cell annotations. Of the normalization techniques, Z normalization best reduced high background signal noise.

## Discussion

Manual identification of cell types in multiplexed imaging data requires significant time and expertise. Noise from imaging artifacts, imperfect segmentation, or tissue processing artifacts can hinder accurate cell-type annotation. Decreasing the time required and increasing the quality of cell type annotations is crucial for conducting robust and reproducible analysis on tissues from large cohorts of subjects. By analysis of cell-type annotation of CODEX multiplexed imaging data that result from several combinations of normalization and clustering approaches provided insight into optimal strategies, summarized in **Figure 6D**.

In general, of the normalization methods we tested (Z, log(double Z), min-max, or arcsinh), Z normalization was the most consistent technique for handling different sources of noise including low intensity signal, high background signal, segmentation noise, and imaging artifacts. Furthermore, Z normalization resulted in accurate identification of both rare and common cell types. Consequently, we recommend Z normalization of values from each marker prior to cell-type identification (**Figure 6Di**).

Accuracy in cell-type identification depended more on normalization technique than it did on the downstream unsupervised clustering algorithms. The algorithms tested, which were the graph-based Leiden, the density-based X-shift with either Euclidian or angular distances (15), and *k*-means clustering, performed similarly, although X-shift clustering with Euclidian distances consistently performed poorly across conditions and annotated standards. Clustering algorithms that resulted in higher numbers of distinct clusters were more accurate in separating distinct phenotypes at the most granular level of cell-type annotation. Furthermore, hand-gating annotations failed to recognize a noise cell type due to cells positive for all markers, image processing artifacts, or tissue artifacts (like folded tissue) and was confounded by segmentation noise such as bleed through of cytokeratin signal into neighboring endothelial cells.

Many granular cell-type definitions that were phenotypic separations (e.g., activated CD4**^+^** T cells *vs.* inactivated CD4^+^ T cells) were inaccurately assigned by all normalization and clustering algorithms. Consequently, irrespective of the clustering algorithm used, settings should be chosen to produce significantly higher number of clusters than cell types expected (**Figure 6** **Dii**). Further detailed annotation should then be done using expression profiles, direct image overlay of cells, and avoiding granular phenotypic cell type calling (**Figure 6** **Diii**).

However, phenotypic markers can be useful once cell types have been established to look at differential expression between the same cell type in different experimental conditions (e.g. PD1 staining on T cells). Also, here we imaged healthy human intestine, whereas in tumor tissues there can be in EMT (Epithelial-Mesenchymal Transition) and MET transition states. This also makes separation of cell types challenging with EMT or MET markers. However, computational analysis such as pseudotime analysis of the transition continuum would be interesting once broader cell types have been established.

With this in mind, selection of antibodies thus plays a significant role in downstream spatial tissue analysis and recognition of cell types. In short, if the research question is to maximize the number of cell types identified to understand the distinct landscape of the tissue, then antibody markers that were restricted to certain cell types (e.g. CHGA-Neuroendocrine) should be selected. On the other hand, phenotypic markers are useful in comparing a certain cell type state in greater detail between experimental conditions.

Beyond making a choice between phenotypic and cell-type markers, antibody clone selection can significantly affect staining quality. Improving the signal-to-noise ratio makes it easier to separate out cell types from one another in unsupervised clustering. Best practices for selecting antibodies can be found in a recent primer (22). Briefly, one should compare available clones, validate with positive and negative controls with both unconjugated and conjugated antibodies, titrate antibody concentration and exposure time, and alter order used within the multiplexed imaging to increase signal-to-noise ratios to improve cell type identification accuracy. While time-consuming, quality assessment of CODEX antibodies will critically impact downstream quantitative results.

In the future, we expect that machine learning-based cell-type annotation transfer models will be built using accurately annotated data (23). This type of model will enable rapid cell-type annotations for replicates or additional samples imaged with a similar imaging panel. This further underscores the necessity for generating an accurate, high-confidence sets of cell-type annotations as training sets.

Additional analytical methods will be needed to address the root causes of additional noise in multiplexed imaging data. As examples, efforts are focused on cell segmentation with generalizable whole-cell segmentation masks trained from a variety of multiplexed imaging data (11). Second, computational methods for correcting small imperfections in segmentation and reassigning signal to the proper cells are in development. Third, amplification approaches within the multiplexed imaging techniques themselves will aid in improving signal-to-noise ratios and elimination of tissue- and imaging-based noise (24, 25). We expect, however, that even after these noise issues are addressed, downstream data analysis will depend on data normalization and cell-type annotation of high-quality reference datasets.

## Data Availability Statement

The datasets presented in this study can be found in online repositories. The names of the repository/repositories and accession number(s) can be found below: HubMAP, HBM977.PCGP.852, HBM575.THQM.284, HBM462.JKCN.863, HBM334.QWFV.953, HBM938.KMNW.825; Dryad https://doi.org/10.5061/dryad.dfn2z352c.

## Author Contributions

Conceptualization: JH, YG, and GPN. Methodology: JH and YG. Formal Analysis: JH, YT, and YG. Writing: JH, YT, YG, and GPN. Funding acquisition: GPN. All authors contributed to the article and approved the submitted version.

## Funding

This work was supported by the U.S. National Institutes of Health (2U19AI057229-16, 5P01HL10879707, 5R01GM10983604, 5R33CA18365403, 5U01AI101984-07, 5UH2AR06767604, 5R01CA19665703, 5U54CA20997103, 5F99CA212231-02, 1F32CA233203-01, 5U01AI140498-02, 1U54HG010426-01, 5U19AI100627-07, 1R01HL120724-01A1, R33CA183692, R01HL128173-04, 5P01AI131374-02, 5UG3DK114937-02, 1U19AI135976-01, IDIQ17X149, 1U2CCA233238-01, 1U2CCA233195-01); the U.S. Department of Defense (W81XWH-14-1-0180, W81XWH-12-1-0591); the U.S. Food and Drug Administration (HHSF223201610018C, DSTL/AGR/00980/01); Cancer Research UK (C27165/A29073); the Bill and Melinda Gates Foundation (OPP1113682); the Cancer Research Institute; the Parker Institute for Cancer Immunotherapy; the Kenneth Rainin Foundation (2018–575); the Silicon Valley Community Foundation (2017-175329 and 2017-177799-5022); the Beckman Center for Molecular and Genetic Medicine; Juno Therapeutics, Inc (122401).; Pfizer, Inc (123214).; Celgene, Inc (133826, 134073).; Vaxart, Inc (137364).; and the Rachford & Carlotta A. Harris Endowed Chair to GN. The funders were not involved in the study design, collection, analysis, interpretation of data, the writing of this article or the decision to submit it for publication. JH was supported by an NIH T32 Fellowship (T32CA196585) and an American Cancer Society - Roaring Fork Valley Postdoctoral Fellowship (PF-20-032-01-CSM).

## Conflict of Interest

GPN received research support from Pfizer, Vaxart, Celgene, and Juno Therapeutics during the course of this work. YG and GPN are inventors on US patent 9909167, granted to Stanford University, that covers some aspects of the technology described in this paper. YG and GPN have equity in and/or are scientific advisory board members of Akoya Biosciences, Inc.

The remaining authors declare that the research was conducted in the absence of any commercial or financial relationships that could be construed as a potential conflict of interest.

## Publisher’s Note

All claims expressed in this article are solely those of the authors and do not necessarily represent those of their affiliated organizations, or those of the publisher, the editors and the reviewers. Any product that may be evaluated in this article, or claim that may be made by its manufacturer, is not guaranteed or endorsed by the publisher.
