# Supplementary material for: Strategies for Accurate Cell Type Identification in CODEX Multiplexed Imaging Data
Source: Front Immunol. 2021 Aug 13;12:727626. doi: 10.3389/fimmu.2021.727626 (PMC8415085; doi:10.3389/fimmu.2021.727626)
Supplement: Supplementary file 1 [file DataSheet_1.pdf]

Supplementary Material

1 Supplementary Data

The data used for this paper is publicly available through the HuBMAP consortia portal.

2 Supplementary Figures and Tables

Supplemental Figures 1-16 are included below

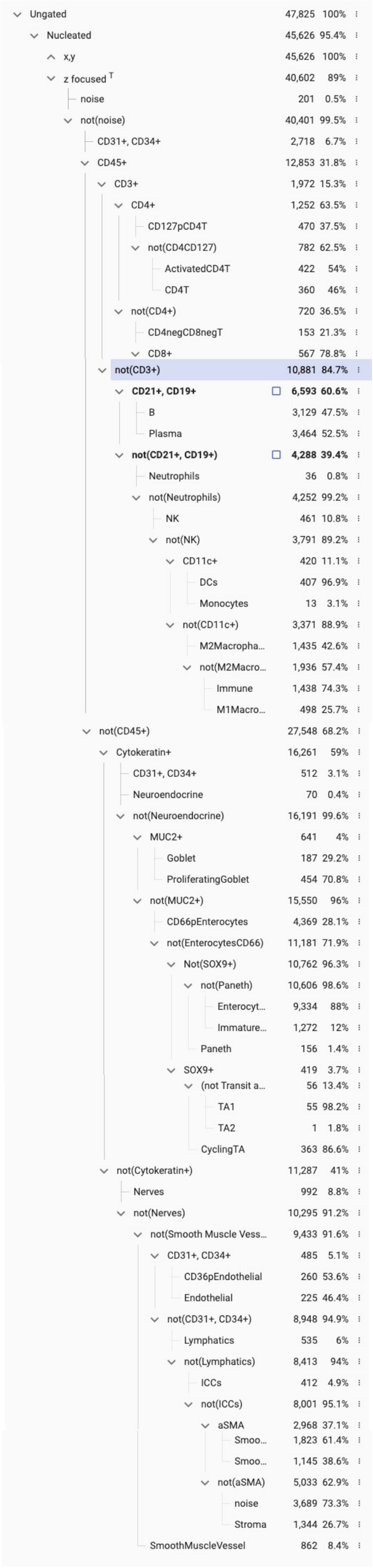

Supplementary Figure 1. Gating strategy for all annotated cell types for the CODEX datasets. A hierarchical, “not” gating approach ensured each cell received a unique cell id.

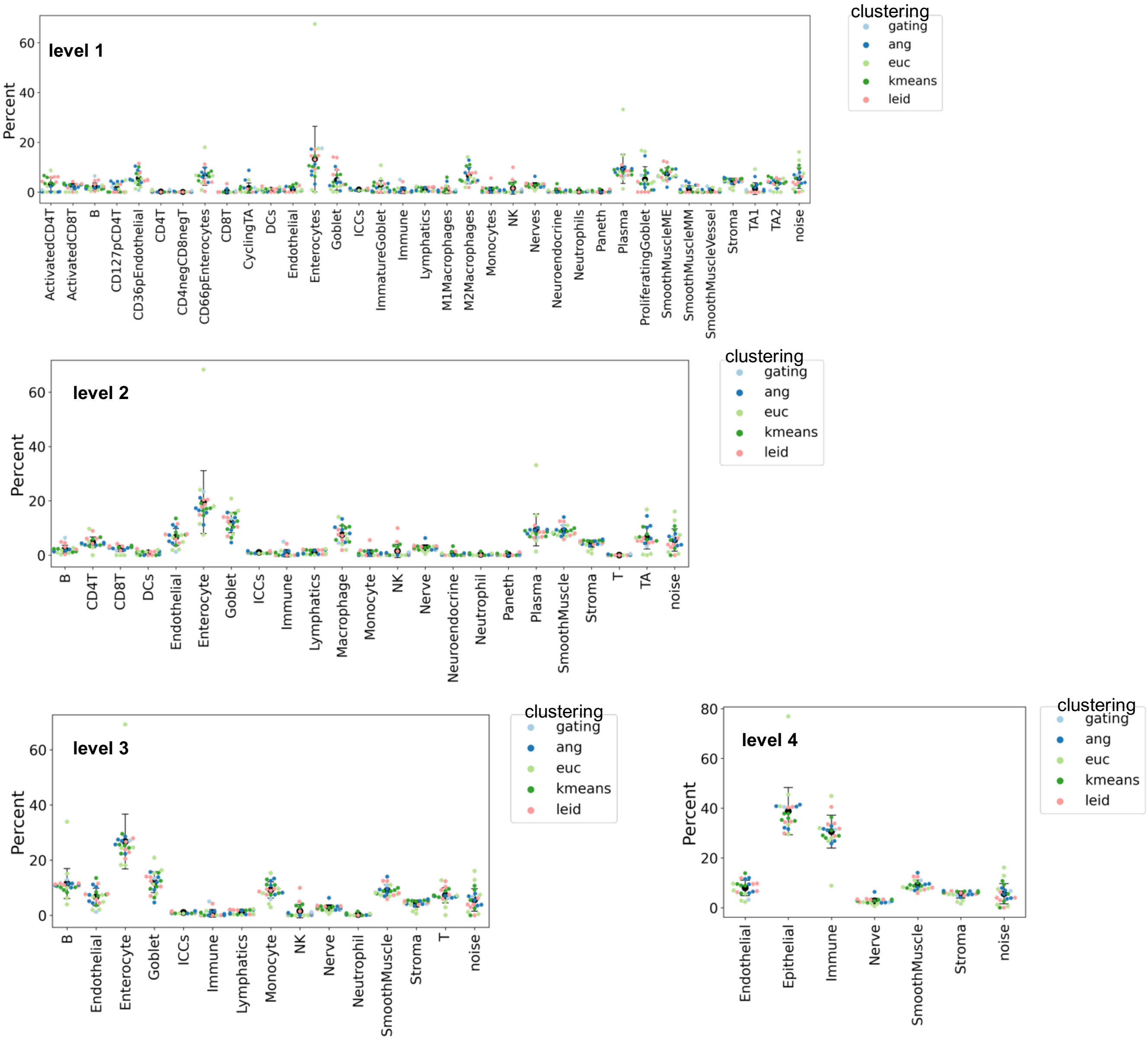
level 1
Percent
0
20
40
60
clustering
gating
ang
euc
kmeans
leid
ActivatedCD4T
ActivatedCD8T
B
CD127pCD4T
CD36pEndothelial
CD4T
CD4negCD8negT
CD66pEnterocytes
CD8T
CyclingTA
DCs
Endothelial
Enterocytes
Goblet
ICCs
ImmatureGoblet
Immune
Lymphatics
M1Macrophages
M2Macrophages
Monocytes
NK
Nerves
Neuroendocrine
Neutrophils
Paneth
Plasma
ProliferatingGoblet
SmoothMuscleME
SmoothMuscleMM
SmoothMuscleVessel
Stroma
TA1
TA2
noise
level 2
Percent
0
20
40
60
clustering
gating
ang
euc
kmeans
leid
B
CD4T
CD8T
DCs
Endothelial
Enterocyte
Goblet
ICCs
Immune
Lymphatics
Macrophage
Monocyte
NK
Nerve
Neuroendocrine
Neutrophil
Paneth
Plasma
SmoothMuscle
Stroma
T
TA
noise
level 3
Percent
0
20
40
60
clustering
gating
ang
euc
kmeans
leid
B
Endothelial
Enterocyte
Goblet
ICCs
Immune
Lymphatics
Monocyte
NK
Nerve
Neutrophil
SmoothMuscle
Stroma
T
noise
level 4
Percent
0
20
40
60
80
clustering
gating
ang
euc
kmeans
leid
Endothelial
Epithelial
Immune
Nerve
SmoothMuscle
Stroma
noise

Supplementary Figure 2. Percentage of cell types across the different levels of granularity, colored by clustering algorithm choice (black data point is mean and error bars indicate standard deviation).

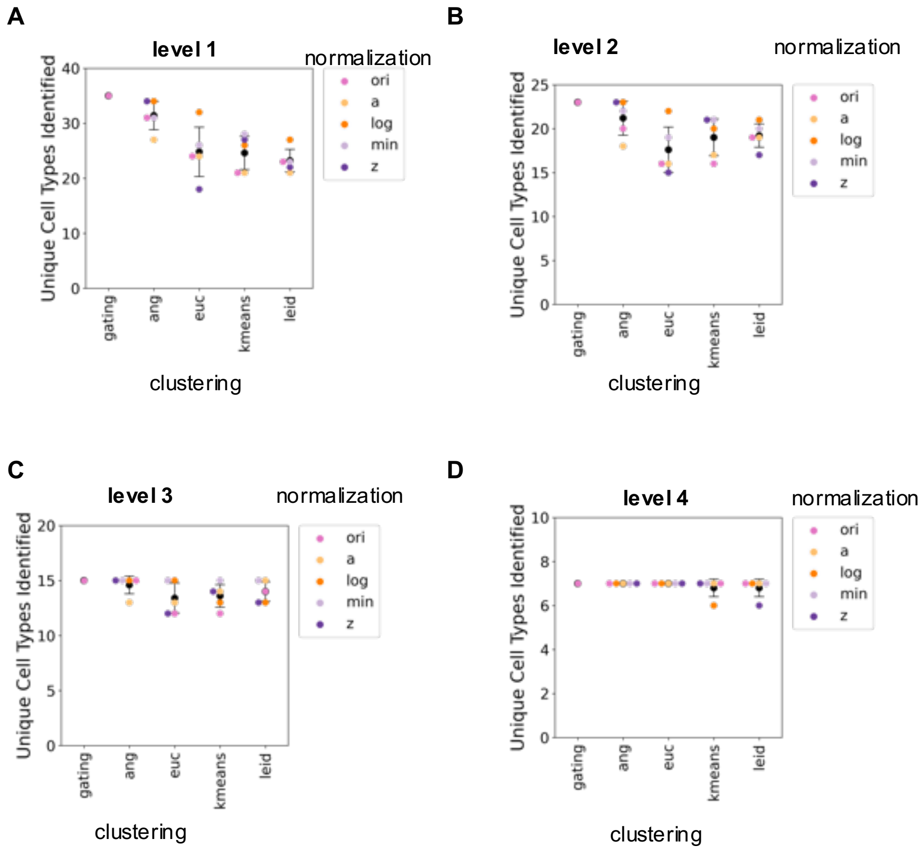

Supplementary Figure 3. Unique number of cell types identified by each combination across **A)** level 1, **B)** level 2, **C)** and level 4 granularity.

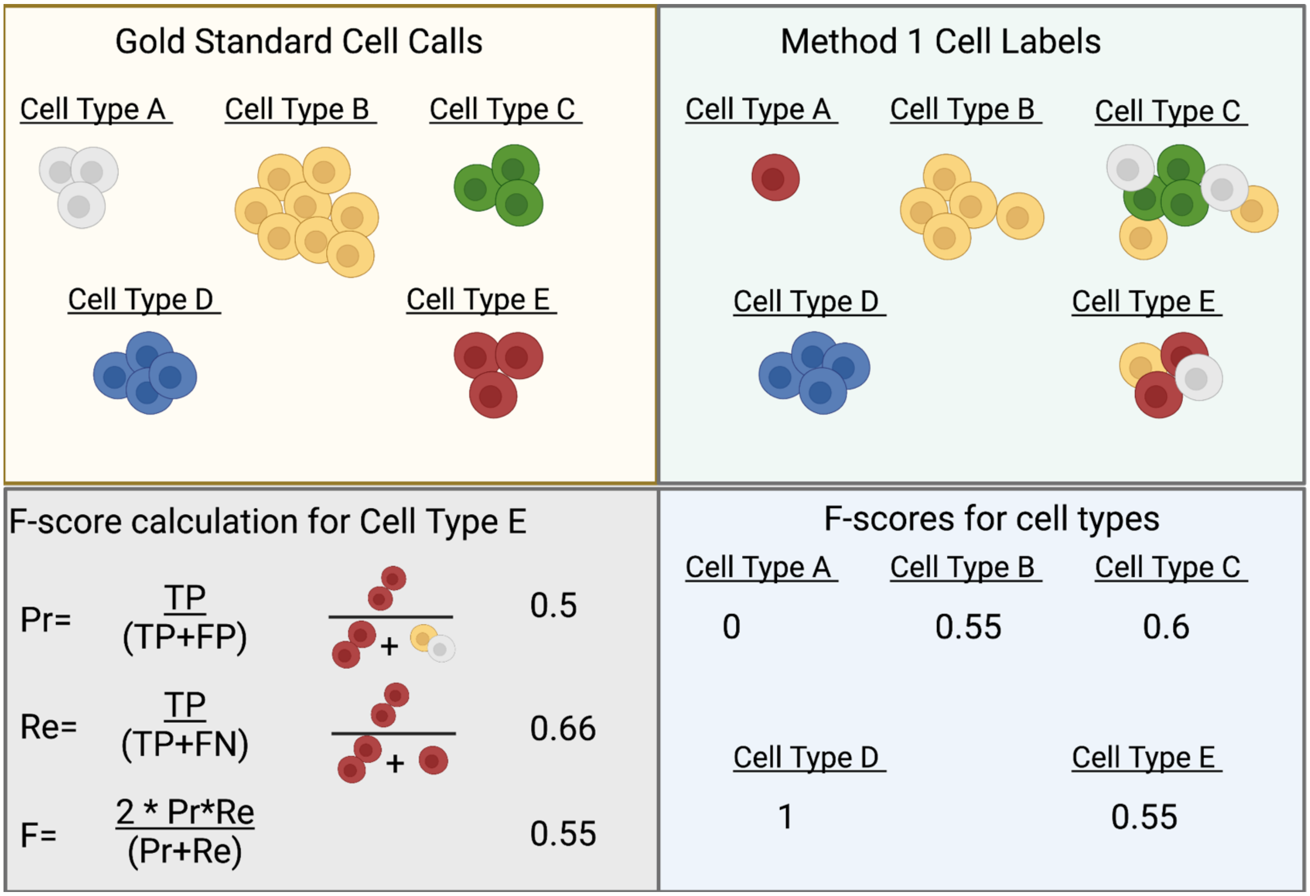

Supplementary Figure 4. Method for calculating F-scores with cell type assignments and representative examples of both imperfect, perfect, and intermediate F-scores with both false negatives and false positives.

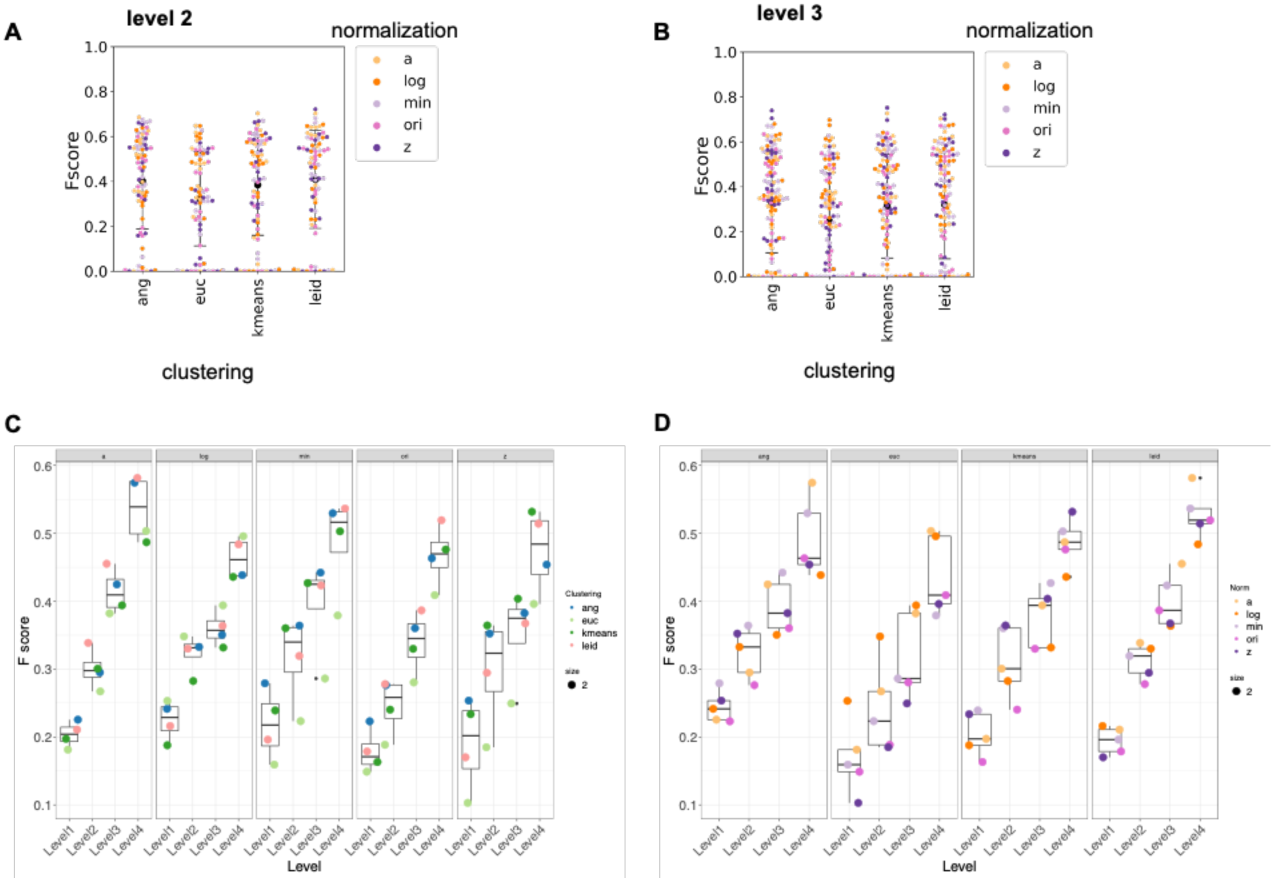

Supplementary Figure 5. F-score comparisons between clustering and normalization technique to hand-gated standard. **A)** Level 2 and **B)** Level 3 F-score averaged for cell types for each combination of normalization technique and clustering algorithm **C**) All level F-score averaged for each combination of normalization and clustering algorithm faceted by normalization technique **D**) All level F-score averaged for each combination of normalization and clustering algorithm faceted by clustering algorithms.

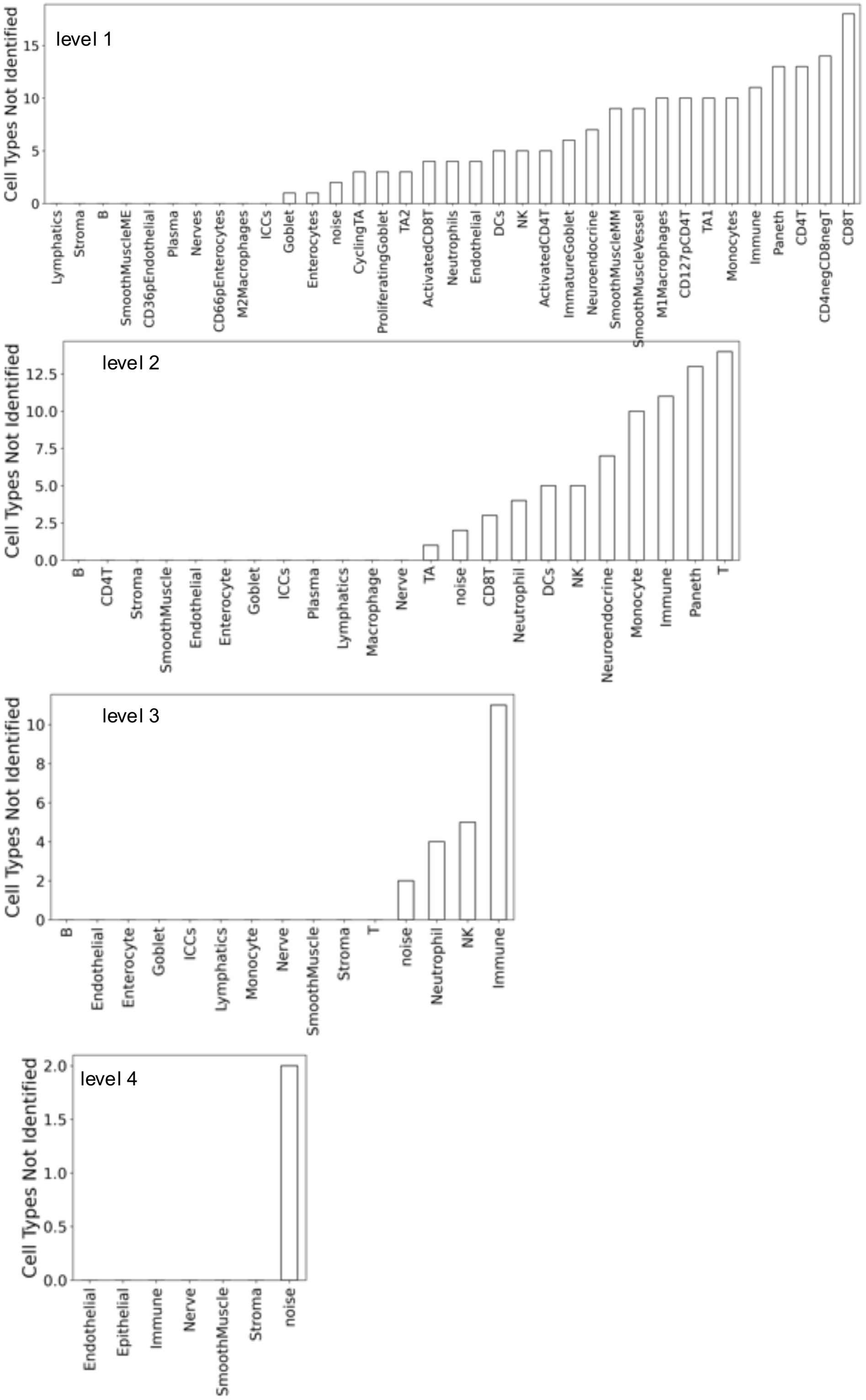

Supplementary Figure 6. Count of unique cell types not identified (summed across all annotations) across the different levels of granularity, sorted by increasing number.

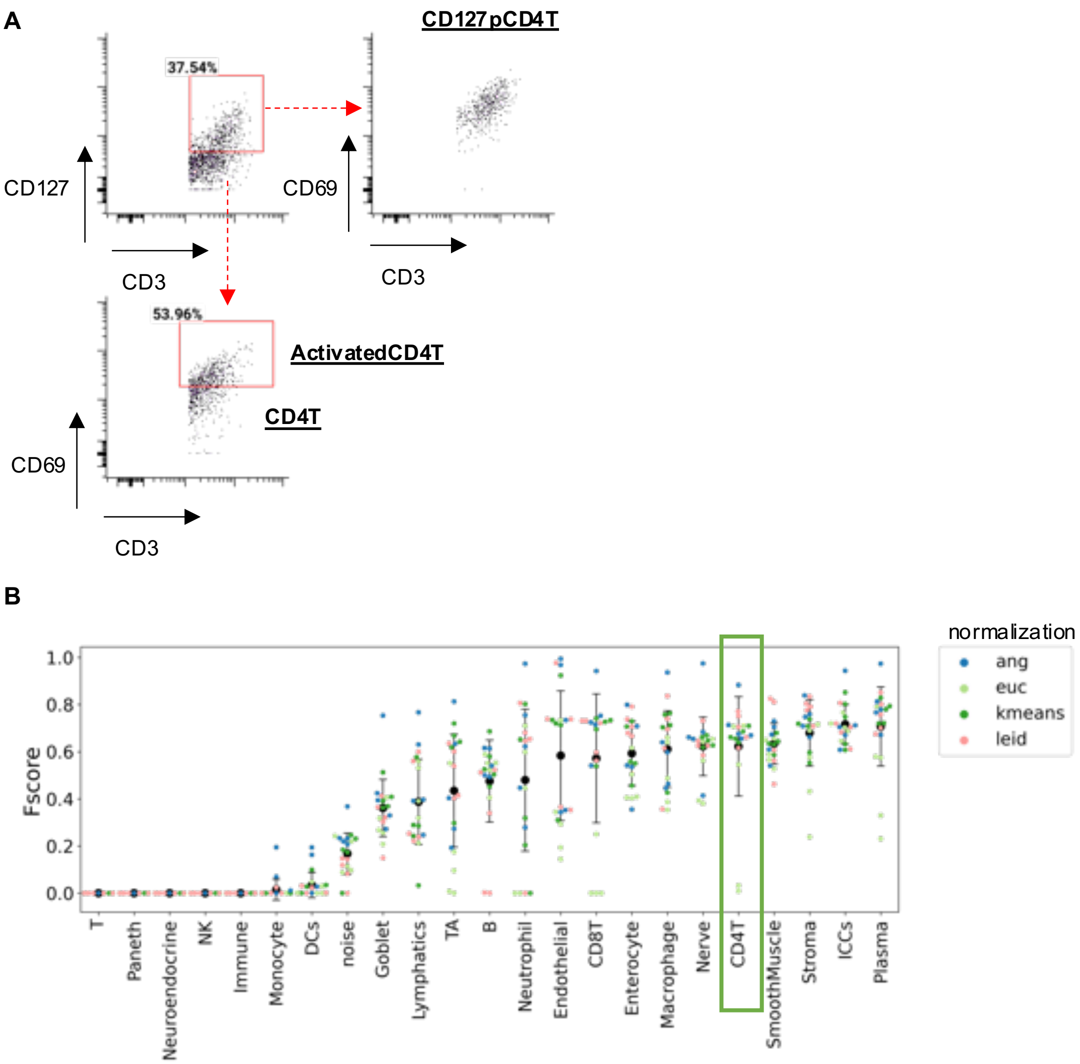

Supplementary Figure 7. High cell type granularity is unreliable as shown with the CD4+ and CD127+ CD4+ T and Activated CD4+ T cell differentiation. **A)** Gating of the three populations where CD127+ are shown in the top right, Activated CD4+ T cells are shown gated in the bottom left within the red gate and CD4+ T cells are shown as negative for CD69+ (1 of 4 regions shown). **B)** Level 2 F-score averaged across cell types for each combination of normalization technique and clustering algorithm compared to over-clustering and image-overlaid annotations (black data point is mean and error bars indicate standard deviation).

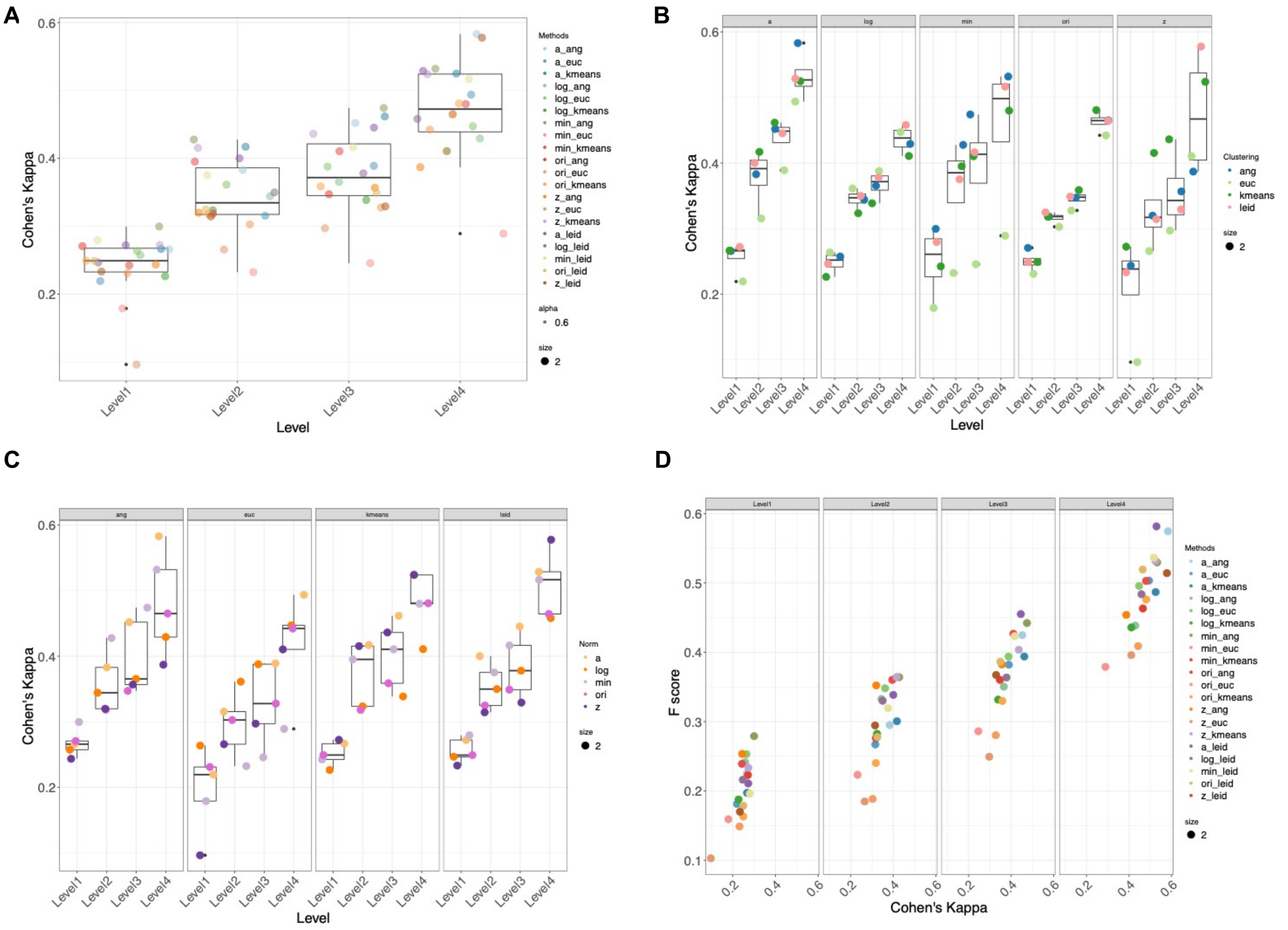

Supplementary Figure 8. Cohen's kappa comparisons between clustering and normalization technique to hand-gated standard. **A)** Cohen's kappa for all levels averaged for each combination of normalization technique and clustering algorithm. **B)** Cohen's kappa for all levels averaged for each combination of normalization technique and clustering algorithm stratified by normalization technique. **C)** Cohen's kappa for all levels averaged for each combination of normalization technique and clustering algorithm stratified by clustering algorithms. **D)** Cross comparison between F score and Cohen's kappa for all levels averaged for for each combination of normalization technique and clustering algorithm**.**

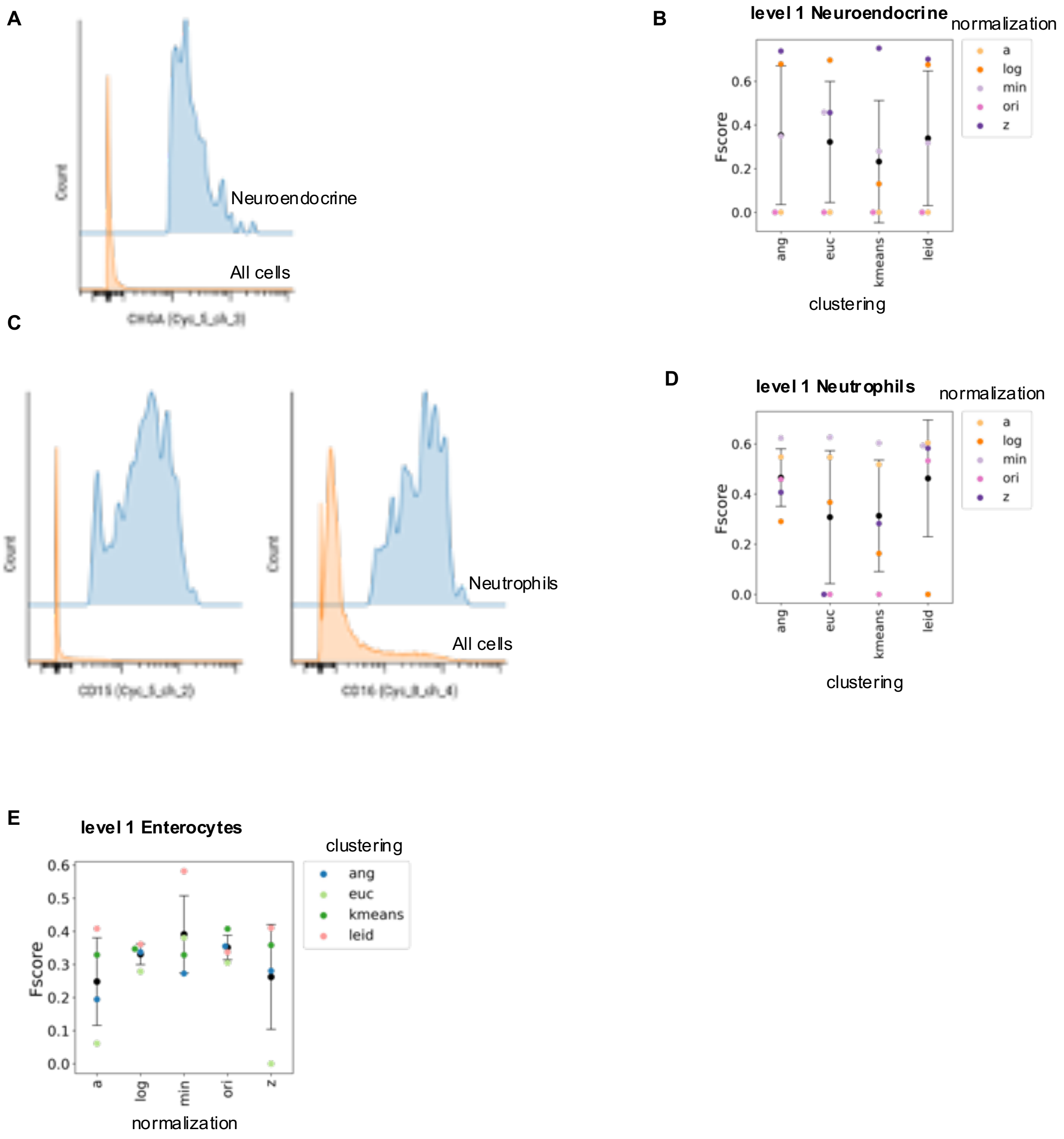

Supplementary Figure 9. Comparisons of clustering and normalization technique at level 1 granularity across different cell types. **A**) Histogram of CHGA fluorescent intensity for all cells in the CODEX dataset (blue = Neuroendocrine-gated cells, orange=all cells). **B**) Level 1 F-scores for Neuroendocrine, pulled out for comparisons of clustering or normalization technique. **C**) Histogram of CD15 and CD16 fluorescent intensity for all cells in the CODEX dataset (blue = Neutrophil-gated cells, orange=all cells). **D-E**) Level 1 F-scores for **D)** Neutrophils and **E)** Enterocytes, pulled out for

comparisons of clustering or normalization technique (black data point is mean and error bars indicate standard deviation).

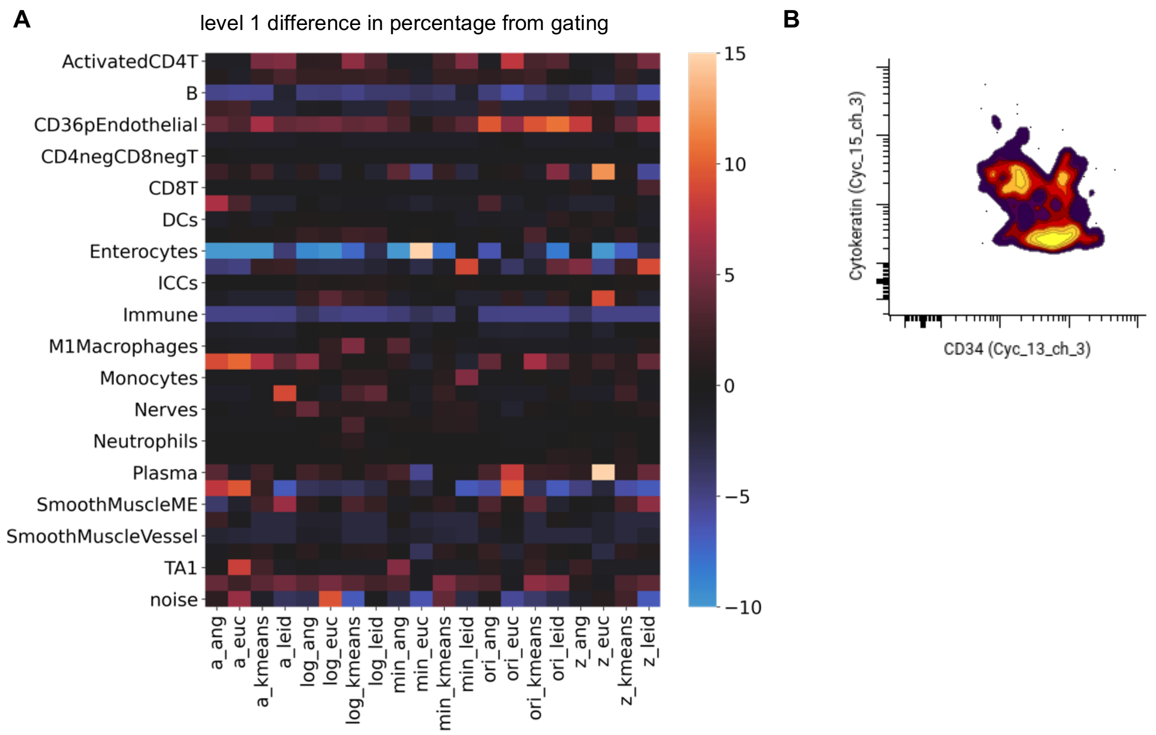

Supplementary Figure 10. Segmentation noise in data is apparent from assignment of Endothelial cell population from hand gated standard. **A)** Heatmap of fold difference between cell type percentages as compared to hand gating annotations for level 1 **B)** The plot of cytokeratin staining levels for individual cells of the mislabeled epithelial cells (blue gate of Figure 4C).

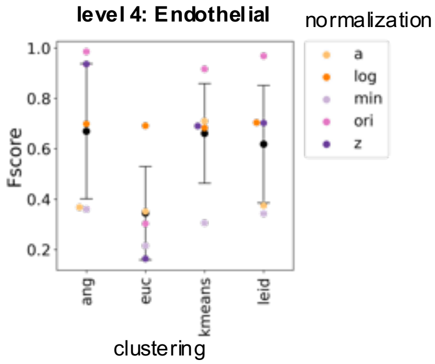

Supplementary Figure 11. Level 4 F-scores for Endothelial, pulled out for comparisons of clustering or normalization technique as compared to over-clustered and image-overlaid annotations.

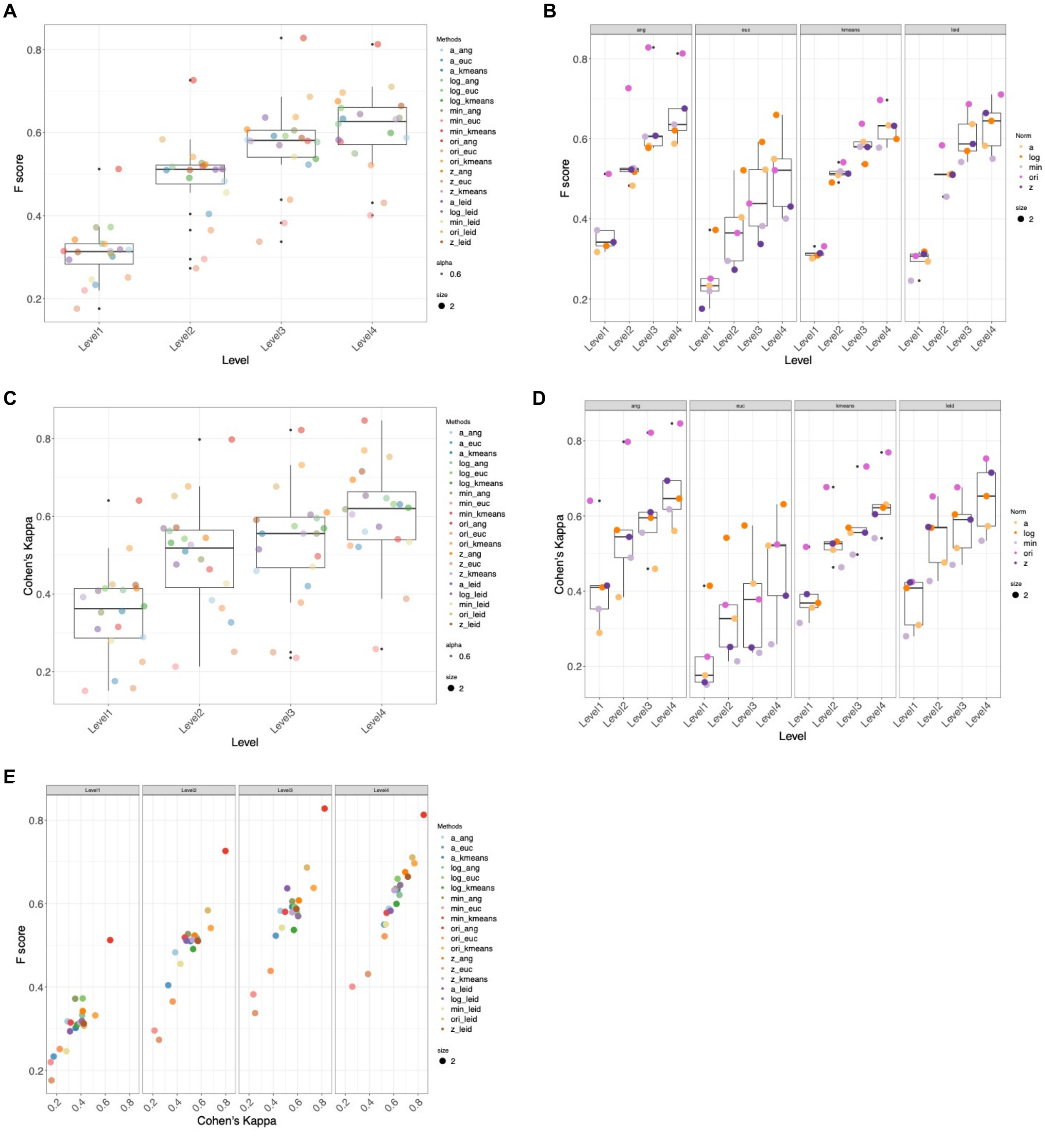

Supplementary Figure 12. Comparing annotations to over-clustered gold standard. **A)** F-scores for all levels averaged for each combination of normalization technique and clustering algorithm. **B)** F-scores for all levels averaged for each combination of normalization technique and clustering algorithm stratified by clustering algorithms. **C)** Cohen's kappa for all levels averaged for each combination of normalization technique and clustering algorithm. **D)** Cohen's kappa for all levels averaged for each combination of normalization technique and clustering algorithm stratified by

clustering algorithms. **E)** Cross comparison between F score and Cohen's kappa for all levels averaged for each combination of normalization technique and clustering algorithm**.**

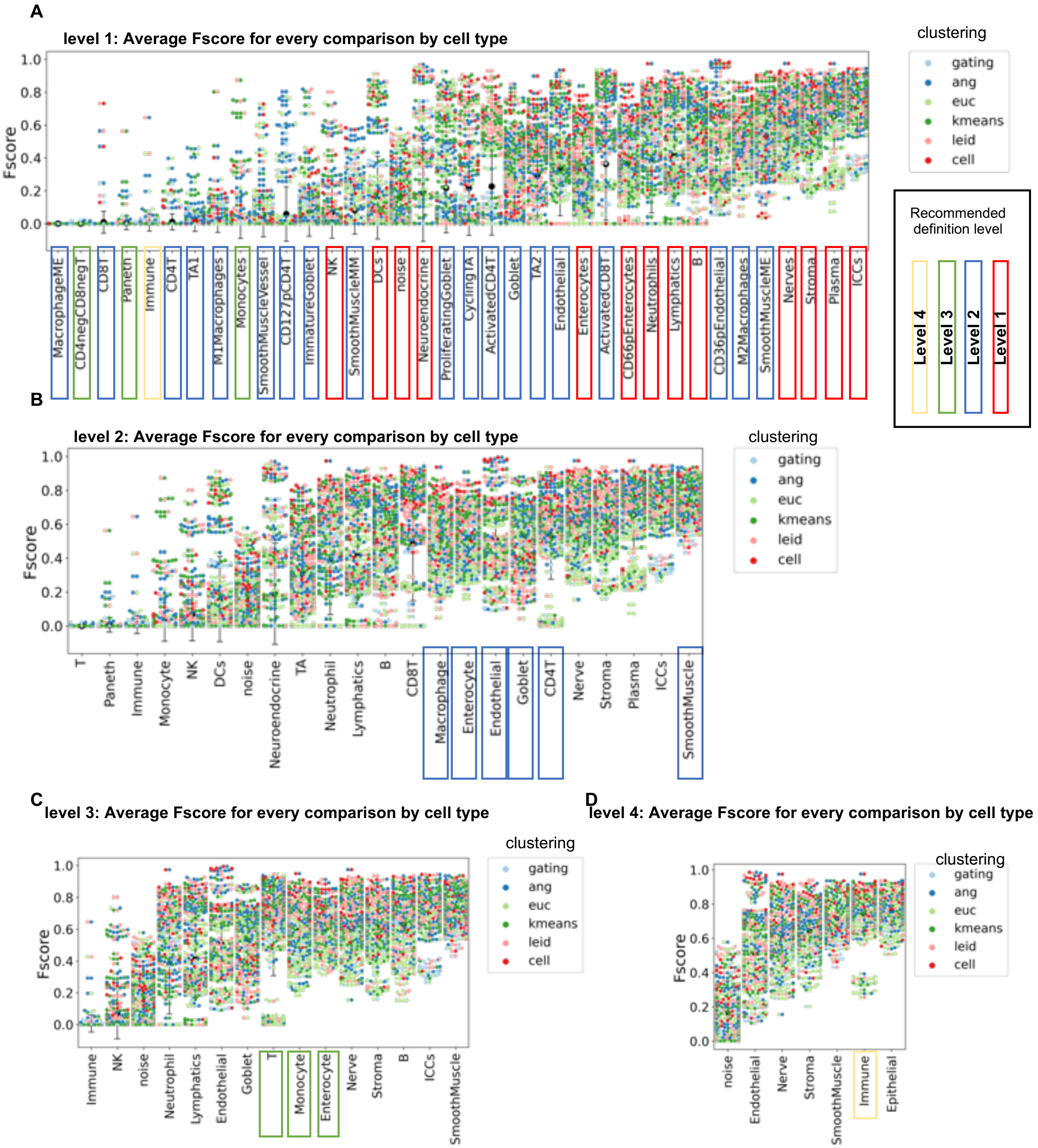

Supplementary Figure 13. Recommended granularity for each cell type population. **A-D)** F-score averaged across cell types for each combination of normalization technique and clustering algorithm compared to all other combinations of normalization technique and clustering algorithm (black data point is mean and error bars indicate standard deviation). Colors of boxes around cell types are coordinated at the level-1 where cell types become reliable (red=level 1, blue=level 2, green = level 3, yellow = level 4)

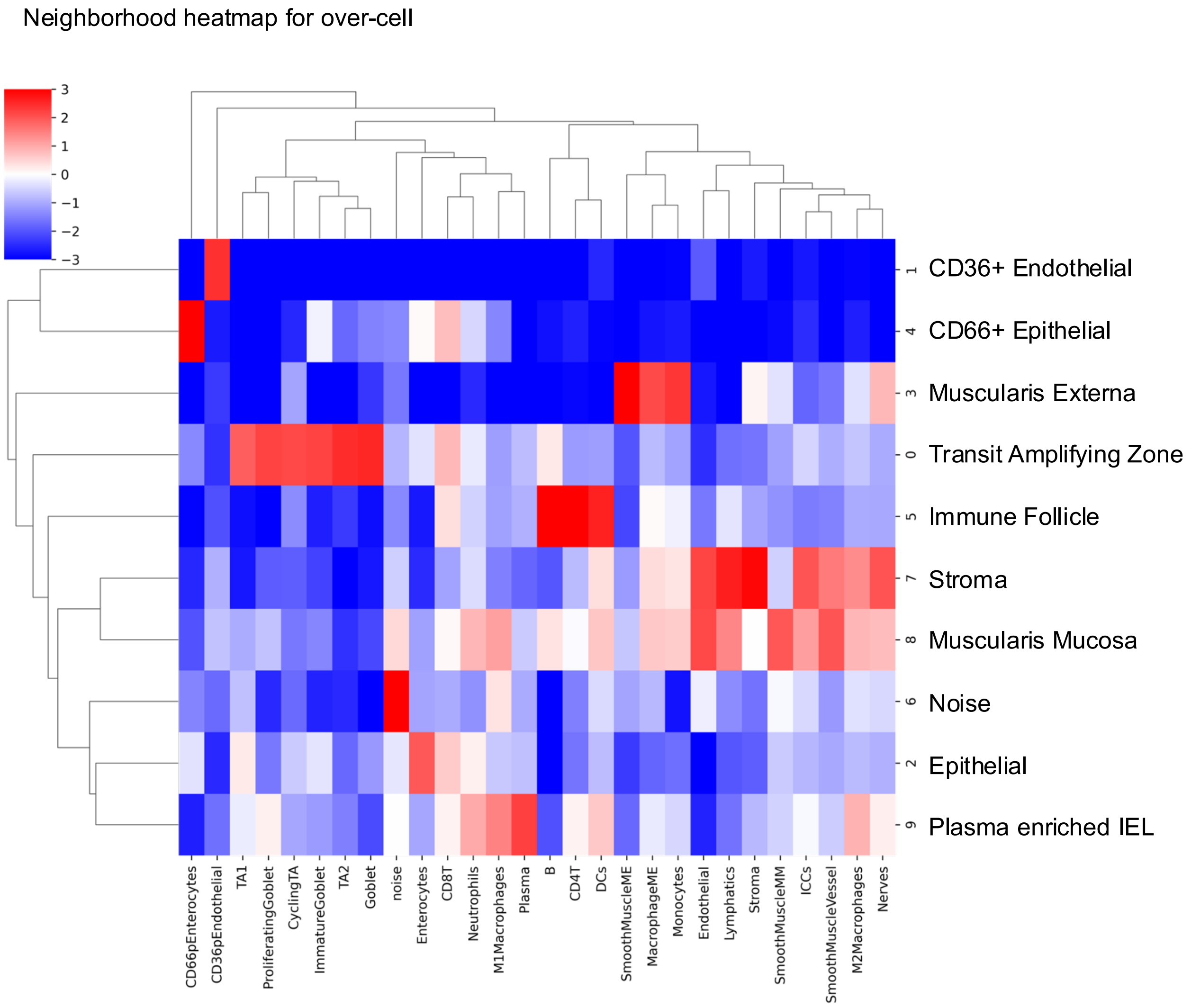

Supplementary Figure 14. Heatmap for cell neighborhoods in the over-clustering and image-overlaid cell type annotations. 10 neighborhoods were annotated for enriched cell types within each neighborhood.

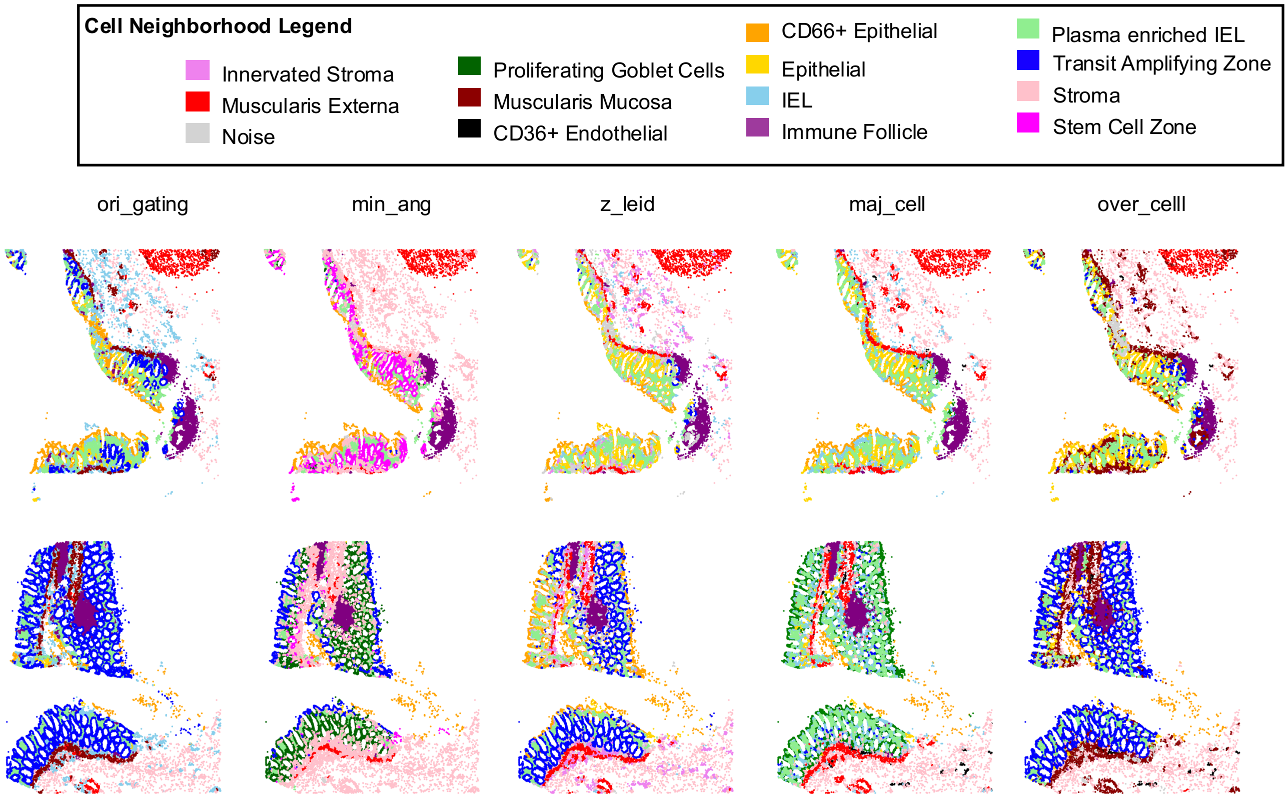

Supplementary Figure 15. Cellular neighborhoods shown for Region 2 and 3 for 5 of the 23 cell-type annotations and colors representing distinct neighborhoods identified.

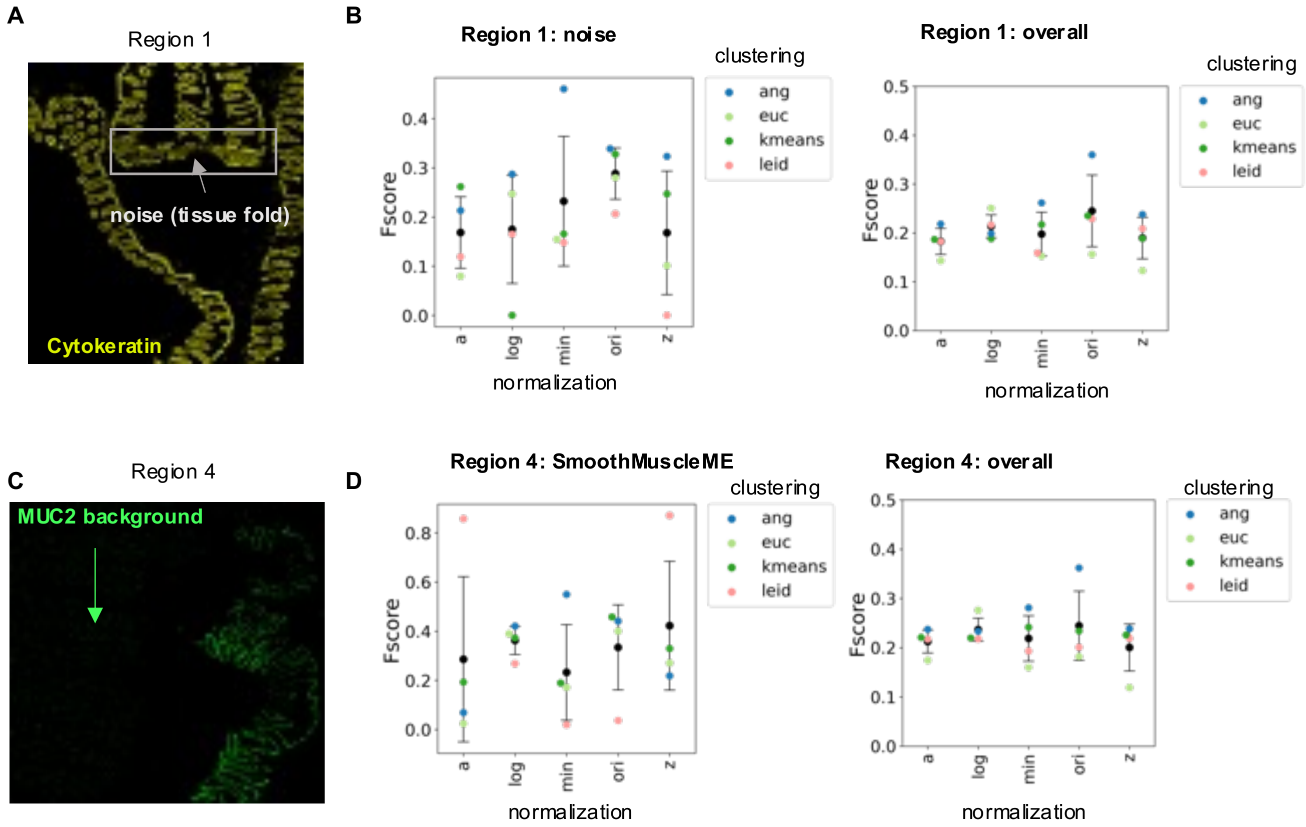

Supplementary Figure 16. Noise identified from neighborhood identification. **A)** Imaging noise indicated in the image where the tissue is folded on itself for Region 1 with Cytokeratin (yellow) staining shown. **B)** Level 1 F-score averages for Region 1 for the noise cell type and averaged across all cell types. **C)** Imaging noise indicated in the image from Region 4 where the MUC2 signal (green) is high in the smooth muscle region. **D)** Level 1 F-score averages for Region 4 for the smooth muscle M.E. cell type and averaged across all cell types (black data point is mean and error bars indicate standard deviation).
